# Key residues regulating von Willebrand factor A1/A2 interactions: insights from molecular dynamics simulations and experimental validation

**DOI:** 10.1016/j.rpth.2025.103267

**Published:** 2025-11-17

**Authors:** Jinhua Fang, Zhiwei Wu, Junxian Yang, Yuming Huang, Aiying Liu, Jiangguo Lin

**Affiliations:** 1 Department of Medical Research, Guangdong Provincial People's Hospital, Guangdong Academy of Medical Sciences, Southern Medical University, Guangzhou, China; 2 School of Medicine, South China University of Technology, Guangzhou, China; 3 Institute of Biomechanics/School of Bioscience and Bioengineering, South China University of Technology, Guangzhou, China; 4 School of Basic Medical Sciences, Southern Medical University, Guangzhou, China

**Keywords:** A1/A2 interaction, AFM, BLI, molecular dynamics simulation, VWF

## Abstract

**Background:**

Von Willebrand factor (VWF), secreted by endothelial cells and megakaryocytes, plays a critical role in hemostasis. Under normal physiological conditions, VWF adopts a globular conformation in which the glycoprotein Ibα–binding site on A1 domain is occluded by the adjacent A2 domain. Studies indicate that exogenous free A2 fragments can improve coagulation function following traumatic brain injury by competitively binding to endogenous VWF-A1, highlighting the therapeutic potential of targeting A1/A2 interaction.

**Objectives:**

This study aimed to identify key residues regulating the A1/A2 interaction at the interface.

**Methods:**

In this study, we used the HADDOCK platform to obtain the optimal conformation of A1/A2 complex and used molecular dynamics simulations to map key binding sites. We conducted multiscale validation using steered molecular dynamics, biolayer interferometry, and atomic force microscopy.

**Results:**

Our results identified 3 pairs of strong interdomain hydrogen bonds at the A1/A2 interface: R1334-E1598, Q1367-V1546, and D1323-R1575. Computational analysis indicated that mutations in both A1_mut (D1323A/R1334A/Q1367A) and A2_mut (V1546A/R1575A/E1598A) reduced the A1/A2-binding affinity and conformational stability. Biolayer interferometry assays confirmed a marked reduction in affinity for A1_mut, while the atomic force microscopy experiment showed only a nonsignificant reduction trend. In contrast, A2_mut significantly disrupted A1/A2 binding in both assays.

**Conclusion:**

By integrating computational prediction with experimental validation, we identified residues D1323, R1334, and Q1367 on A1, along with V1546, R1575, and E1598 on A2, as key residues that maintain the A1/A2 interaction. These findings enhance our understanding of the VWF-A1/A2 interaction mechanism and provide novel molecular targets and a theoretical basis for developing targeted therapies for associated disorders.

## Essentials

• The VWF A2 domain occludes the GPIbα-binding site on the VWF A1 domain.
• Key residues of A1/A2 were identified by computational prediction and validated by SMD, BLI, and AFM.
• Three key residue pairs form a strong hydrogen bond network crucial for the A1/A2 interaction.
• Mutations disrupted the A1/A2 binding, highlighting these residues as potential therapeutic targets.

## 1 Introduction

von Willebrand factor (VWF) is a multimeric glycoprotein known for its adhesive properties [1]. It is primarily synthesized and secreted by megakaryocytes and vascular endothelial cells. Following synthesis and processing, most VWF molecules assemble into multimers of various sizes through head-to-head and tail-to-tail connections. These multimers are stored in platelet α-granules and Weibel–Palade bodies within endothelial cells [2]. Upon vascular injury, VWF is rapidly released and exposed at the damaged site, where it binds specifically to the glycoprotein (GP)Ibα on the platelet membrane. This interaction facilities the initial adhesion and aggregation of platelets on the damaged vessel wall, thereby initiating the hemostatic process. Additionally, VWF serves as a carrier for coagulation factor (F)VIII, stabilizing its levels in the plasma [3]. Abnormalities in VWF function—such as reduced quantity, structural defects, or functional disorders—can lead to von Willebrand disease (VWD), a common inherited bleeding disorder characterized by varying degrees of hemostatic and coagulation dysfunction [4,5].

Under normal physiological conditions, VWF adopts a loosely collapsed, globular conformation that prevents platelet binding. In this state, its A1 domain is shielded by adjacent functional regions, such as the D'D3, A2, and A3 domains [6,7]. This self-inhibitory mechanism prevents premature activation of VWF and unnecessary platelet aggregation under normal blood flow conditions. However, upon vascular injury, this equilibrium is disrupted as VWF binds to exposed subendothelial collagen, securely anchoring itself at the site of damage. Subsequently, under high hemodynamic shear forces, VWF undergoes a significant conformational change: its coiled structure progressively unfolds, revealing the cryptic A1 domain. This alteration alleviates steric hindrance at the GPIbα-binding site of the A1 domain, allowing it to recruit and activate circulating platelets, thereby initiating adhesion and aggregation at injury sites [8,9].

The A2 domain of VWF plays a pivotal role in regulating the interaction between VWF and platelets. Experimental evidence demonstrates that A1A2A3 exhibits significantly weaker affinity to GPIbα than the isolated A1 domain [10]. Deletion of the A2 domain markedly enhances ristocetin-induced binding of VWF to platelet GPIbα [11]. The addition of exogenous A2 domain fragments substantially reduces platelet adhesion, indicating that the A2 domain functions as an inhibitor regulator of the A1-mediated platelet binding [12]. Conversely, the A1 domain inhibits the cleavage of A2 by ADAMTS-13 (a disintegrin and metalloprotease with thrombospondin type 1 motif, 13), while the binding of VWF-A1 to GPIbα enhances the cleavage efficiency [13]. The redox state of the C-terminal disulfide bond (C1669-C1670) within the A2 domain critically regulates the A1-A2 interaction. A study proposes that reduced form of A2 (C1669A/C1670A) binds more tightly to A1, thereby strengthening its inhibition of A1-GPIbα binding [14]. However, research by the Tischer et al. [10] team in 2023 suggests that reduction (C1669S/C1670S) destabilizes the A2 domain, disrupting the normal interaction network among the A1, A2, and A3 domains and slightly enhancing the GPIbα-binding capacity of the A1A2A3 fragment. Additionally, methionine oxidation has been proposed to impair the folding stability the A2 domain and alter its interaction interface with A1. This alteration exposes the GPIbα-binding site in A1, diminishing the inhibitory effect of the A2A3 region on A1-mediated platelet adhesion and ultimately increasing VWF-A1 binding to GPIbα [[15], [16], [17]]. Therapeutically, Xu et al. [18] demonstrated in a murine model that exogenous free A2 domain fragments ameliorate coagulation dysfunction following traumatic brain injury by interacting with endogenous VWF-A1. These findings position the free A2 fragment as a promising therapeutic agent for thrombotic disorders. Therefore, a deeper understanding of the interaction mechanisms and regulatory networks between the VWF-A1 and A2 domains is essential, to not only elucidate their roles in physiological and pathological processes but also identify critical molecular targets for developing novel targeted therapies for platelet-mediated diseases.

Despite these insights, the lack of the crystal structure of the VWF-A1/A2 complex obscures key details regarding its binding interface. To bridge this gap, computational approaches such as molecular docking have been used. Aponte-Santamaría et al. [19] used PatchDock and FireDock to generate 6 potential A1/A2 conformational models, suggesting that the A2 domain inhibits A1 function by occluding its β_3_-sheet [19]. Similarly, Karoulia et al. [20] predicted 3 distinct A1/A2 models using HEX docking, identifying residues E1549 and E1554 in model A; E1640 in model B; and E1511, E1519, E1522, and D1663 in model C as potentially critical for A1/A2 interaction [20]. However, these predictions are computationally derived and lack experimental validation. In this study, we performed molecular docking using HADDOCK platform to derive the optimal conformation of A1/A2 complex and predicted the key residues at its binding interface through equilibrium molecular dynamics (MD) simulation. Corresponding mutants were then constructed, and the importance of these residues for maintaining the A1/A2 interaction was experimentally validated using MD simulations, biolayer interferometry (BLI), and atomic force microscopy (AFM). Notably, we identified D1323, R1334, Q1367, V1546, R1575, and E1598 as key mediators of this interaction, with our findings verified through experimental approaches.

## 2 Materials and Methods

### 2.1 Materials

Streptavidin-coated biosensors were obtained from Sartorius. Biocytin was obtained from Sigma-Aldrich. Mouse anti-6×His monoclonal antibody was obtained from Abcam. AFM probes were obtained from Bruker MLCT. Plasmids for recombinant VWF-A1 (amino acids Q1238-D1472), VWF-A2 (amino acids M1473-G1672), and their respective mutants were constructed through homologous recombination using full-length VWF plasmid (MiaoLingBio) as the template. The recombinant VWF-A1 and VWF-A2 polypeptides, each containing a His tag at their N-terminus, were expressed in HEK293F cells. The culture medium was collected and purified using a Ni^2+^-Hi-Trap–chelating column (GE Healthcare). Purity of the proteins was confirmed by sodium dodecyl sulfate polyacrylamide gel electrophoresis.

### 2.2 Construction of the VWF-A1/A2 complex

The structures of the VWF-A1 domain (amino acid sequence D1261-T1468, code 1AUQ) [21] and the VWF-A2 domain (amino acid sequence M1495-S1671, code 3GXB) [22] were obtained from the Protein Data Bank (PDB). The CPORT (Consistent Prediction of Interface Residues in Transient Complexes) server was used to predict positive and negative residues at the interface between the VWF-A1 and VWF-A2 domains. These residues served as spatial restraints for flexible molecular docking using the HADDOCK 2.2 platform [23]. The docking procedure generated 39 binding poses, which were systematically classified into 8 distinct clusters based on HADDOCK scoring metrics and root mean square deviation (RMSD) values. The composite HADDOCK score integrates 5 critical parameters: van der Waals energy, electrostatic energy, desolvation energy, restraint violation energy, and buried surface area. Lower scores indicate more favorable binding conformations. To refine the selection process, visual inspections were performed using visual molecular dynamics (VMD) software. Three clusters that met the structural criterion—occlusion of the GPIbα-binding site on VWF-A1 by the adjacent VWF-A2 domain—were prioritized for further analysis. Subsequently, these 3 clusters underwent rigorous energy minimization followed by 20-nanosecond equilibration simulations using the Nanoscale MD package. This MD protocol enabled the identification of the optimal conformation of the complex for subsequent experiments.

### 2.3 System setup and MD simulation

System setup was conducted using VMD software. The selected protein complex was solvated in a TIP3P water box extending 1.5 nm from the molecular surface, with 0.154 M NaCl added to mimic physiological ionic concentrations and maintain system electroneutrality. The CHARMM27 all-atom force field and periodic boundary conditions were used for the MD simulation. After performing 15,000 steps of energy minimization, the system enters a 50-ns equilibrium phase under isothermal-isobaric ensemble conditions using Nanoscale MD software. Temperature was maintained at 310 K using Langevin dynamics, and pressure was controlled at 1 atmosphere using the Langevin piston method. Key residues in the A1/A2 interaction were predicted by analyzing hydrogen bonds and salt bridges at the binding interface during equilibrium. Subsequently, these predicted key residues on the A1 and A2 domains were mutated to alanine, resulting in the A1 mutant complex (designated A1_mut complex) and the A2 mutant complex (designated A2_mut complex). These 2 complexes underwent 3 independent energy minimization and equilibrium simulations to further examine the effects of these mutations on the A1/A2 interaction.

To assess the mechanical stability of the A1/A2 interaction, we performed constant velocity stretching simulations, the force-ramp model in steered molecular dynamics (SMD) simulation. The stable conformation after 50-nanosecond equilibrium was used as the initial setup for the SMD simulation. In the SMD simulation, the C-terminal Cα atom of the A2 domain was fixed, while the N-terminal Cα atom of the A1 domain was pulled at a constant velocity of 5 Å/ns along the line connecting the steered and fixed atoms. The steered and dummy atoms were linked by the virtual spring with a spring constant of 69.48 pN/Å. The rupture force was determined from the peak in the force-time curve obtained during the 25-nanosecond SMD simulation.

### 2.4 Data analysis for MD simulations

All analyses were performed with VMD tools. The Cα RMSD was calculated to assess conformational stability. The number of hydrogen bonds, the buried solvent accessible surface area (SASA), and binding energy were evaluated to assess the binding ability between A1 and A2. A hydrogen bond was defined when the donor-acceptor distance was < 3.5 Å, and the angle was < 30°. A salt bridge was defined when the distance between the oxygen atom of an acidic residue and the nitrogen atom of a basic residue was < 4 Å. The survival rates of hydrogen bonds and salt bridges were analyzed to predict key residues at the A1/A2-binding interface. Dissociation times and rupture forces were extracted from the force-time curve obtained during the SMD simulations.

### 2.5 BLI assays

Binding kinetics between A2 and A1 mutants were measured using BLI on an Octet R8 instrument (Sartorius) under controlled conditions (30 °C, 1000 rpm). Biotinylated A2 was diluted to 10 μg/mL in assay buffer (phosphate-buffered saline containing 0.02% Tween-20 and 0.1% bovine serum albumin [BSA]) and immobilized onto streptavidin-coated biosensors (Octet SA Biosensors; Sartorius) via biotin-streptavidin interactions for 300 seconds. The sensors were then quenched with 10 μg/mL biocytin for 180 seconds. After a 120-second baseline stabilization in assay buffer, the sensors were exposed to serial dilutions of A1 (125-4000 nM) for 60 seconds during the association phase, followed by dissociation in assay buffer for 120 seconds. Biosensors were regenerated after each cycle using 10 mM glycine (pH 2). Data were processed with Octet Analysis Studio software (Sartorius), and binding curves were globally fitted to a 1:1 Langmuir binding model to calculate the affinity constant (*K*_D_).

### 2.6 AFM assays

AFM was used to quantitatively characterize the dynamics of interdomain interactions between VWF-A1 and VWF-A2, specifically focusing on adhesion frequency and rupture force measurements. Mouse anti-His monoclonal antibody (15 μg/mL) was adsorbed onto both polystyrene petri dishes and AFM cantilever tips overnight at 4 °C. Nonspecific binding sites were blocked by treating all surfaces with 2% BSA for 1 hour. Following this surface preparation, the AFM tips were conjugated with VWF-A2 (15 μg/mL), while the petri dishes were functionalized with either wild-type (WT) VWF-A1 (15 μg/mL) or its variants. During force spectroscopy measurements, cantilevers contacted with substrate surfaces at an approach-retraction speed of 200 nm/s in BSA-containing solution (2%). This cyclic contact procedure (100 cycles per measurement point) generated force-time curves, which were subsequently analyzed using dedicated AFM software. Five points were measured for each substrate, and at least 3 independent substrates were measured. Binding events were identified through characteristic rupture signatures in the force curves, with adhesion frequency calculated as the ratio of binding events to total contact cycles. The rupture forces were quantified by measuring the maximum force required to dissociate the VWF-A1/A2 complex during cantilever retraction.

### 2.7 Statistical analysis

Data are presented as mean ± SEM from at least 3 independent experiments. The distribution of the hydrogen bond number was fitted with a Gaussian function. Statistical comparisons of mean values among multiple groups were assessed using 1-way analysis of variance (anova) and 2-way anova with Dunnett test for multiple comparisons, as indicated in figure legends. A *P* value of <.05 was considered statistically significant. All analysis was conducted using Prism 9 software (GraphPad).

## 3 Results

### 3.1 Optimal structure of the VWF-A1/A2 complex

**Figure 1:**
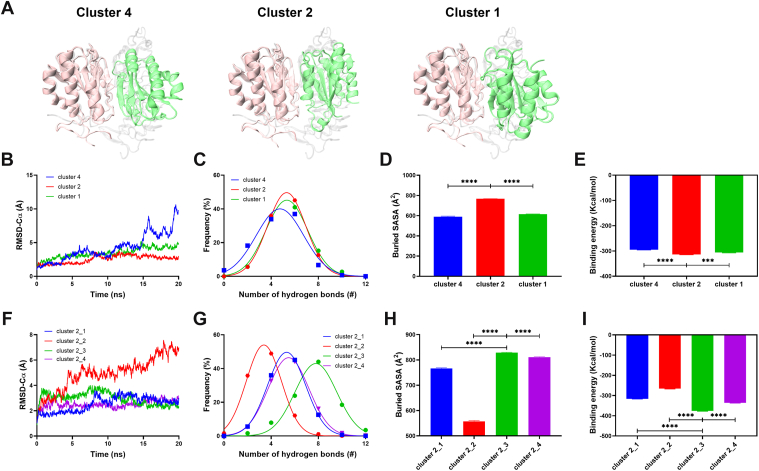
Screening of the docking conformation of A1/A2 complexes. (A) Molecular docking–derived structures of cluster 4, cluster 2, and cluster 1. The von Willebrand factor (VWF)-A1/A2 complex conformation (orange, VWF-A1 domain; green, VWF-A2 domain) is superimposed on the VWF-A1/GPIbα complex (1SQ0, white base layer). Comparative analysis of cluster 4, cluster 2, and cluster 1 during 20-nanosecond equilibrium: time courses of RMSD-Cα (B), distribution of hydrogen bonds number (C), buried SASA (D), and binding energy (E). Detailed characterization of 4 representative poses in cluster 2: time courses of RMSD-Cα (F), distribution of hydrogen bonds number (G), buried SASA (H), and binding energy (I) of 4 poses in cluster 2. All data are shown as the mean ± SEM. One-way anova with Dunnett test for multiple comparisons was used to test statistical significance. RMSD, root mean square deviation; SASA, solvent accessible surface area. ∗∗∗*P* < 0.001; ∗∗∗∗*P* < .0001.

Using HADDOCK docking, 39 possible conformations for VWF-A1/A2 complexes were obtained, which were grouped into 8 clusters. According to the principle that VWF-A2 should shield the binding site of VWF-A1 and platelet GPIbα [6], as well as the HADDOCK score, we overlapped the A1/A2 structural conformations with the A1/GPIbα structure (PDB code 1SQ0) [24] to identify 3 candidate conformations (clusters 4, 2, and 1) (Figure 1A). The highest scoring pose in each cluster was then subjected to a 20-nanosecond equilibrium simulation. Among these, cluster 4 exhibited poorer conformational stability, as indicated by its fluctuating RMSD value (Figure 1B). In contrast, cluster 2 demonstrated the most stable RMSD throughout the simulation (Figure 1B), accompanied by the greatest number of hydrogen bonds (Figure 1C), the largest buried SASA (Figure 1D), and the lowest binding energy (Figure 1E), making it the superior choice. Comparison with the other 5 clusters (clusters 3, 6, 8, 7, and 5) reaffirmed that cluster 2 was the best conformational choice (Supplementary Figure 1). Further examination of the 4 poses within cluster 2 led to the exclusion of cluster 2_2 due to its poor conformational stability (Figure 1F). Cluster 2_3, however, exhibited remarkable stability, with stable RMSD values (Figure 1F), the highest number of hydrogen bonds (Figure 1G), the largest buried SASA (Figure 1H), and the lowest binding energy (Figure 1I). These attributes highlight the unique stability and strong interaction profile of this cluster. Thus, cluster 2_3 was designated as the optimal structure and served as the initial WT structure for subsequent equilibrium simulations and mutation studies.

### 3.2 Residues D1323, R1334, and Q1367 on VWF-A1 and V1546, R1575, and E1598 on VWF-A2 were predicted to be key residues for VWF-A1/A2 interaction

**Figure 2:**
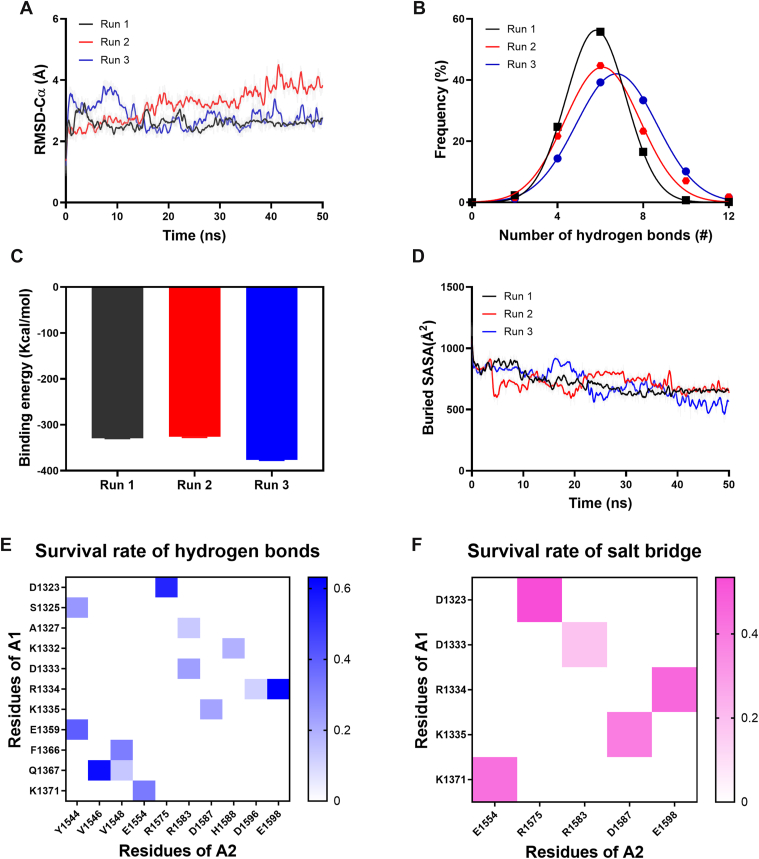
Variations of structural stability and the interaction of the von Willebrand factor (VWF)-A1/A2 complex during three 50-nanosecond equilibrium simulations. (A) Time course of root mean square deviation (RMSD)-Cα were steady in 3 runs. (B) The frequency of hydrogen bonds number was fitted by Gaussian distribution. The mean binding energy (C) and buried solvent accessible surface area (SASA) (D) of 3 runs are shown. The heat maps of hydrogen bonds (E) and salt bridges (F) visually present the spatial distribution characteristics of intermolecular interactions.

Cluster 2_3 was further subjected to 3 independent 50-nanosecond equilibrium simulations to identify key residues at the A1/A2-binding interface. The RMSD time course across these simulations remained stable after 50 nanoseconds (Figure 2A), implying that the conformation reached an equilibrium state. Additionally, the number of hydrogen bonds across the 3 simulations conformed to the Gaussian distribution and was relatively close (Figure 2B), and the binding energy and buried SASA were also comparable across 3 runs (Figure 2C, D).

To analyze the hydrogen bonds and salt bridges formed at the binding interface, we calculated the survival rates of each hydrogen bond and salt bridge at the binding interface during 3 equilibrium simulations (Tables 1 and 2). Heat maps were generated for hydrogen bonds and salt bridges with an average survival ratio >10% (Figure 2E, F). The heat maps indicated that VWF-A1/A2 binding predominantly localized in 2 regions: the β_3_ sheet of VWF-A1 binding to the α_3_ helix of VWF-A2 and the α_3_ helix of VWF-A1 interacting with the β_3_ sheet of VWF-A2. This observation aligns with earlier speculation that VWF-A2 masks the β_3_ sheet of VWF-A1 [19]. A total of 13 pairs of hydrogen bonds with a survival rate exceeding 10% were identified at the A1/A2-binding interface. Notably, the top 3 pairs were R1334-E1598, Q1367-V1546, and D1323-R1575, all exhibiting survival rates >50% (Table 1). In addition, D1323 formed the strongest salt bridge with R1575, while R1334 formed a second strong salt bridge with E1598 (Table 2). Collectively, these findings suggest that residues D1323, R1334, and Q1367 from VWF-A1 and V1546, R1575, and E1598 from VWF-A2 are critical for the A1/A2 interaction and were selected for subsequent mutational studies.

**Table 1:** The hydrogen bonds on the interface of the A1/A2 complex with an average survival ratio exceeding 10%.

No.	A1	A2	Survival rate (%)			Average (mean ± SEM)
			Run 1	Run 2	Run 3	
1	R1334	E1598	79.3	59.1	51.1	63.2 ± 8.4
2	Q1367	V1546	78.1	74.8	27.9	60.3 ± 16.2
3	D1323	R1575	67.7	5.7	88.7	54.0 ± 24.9
4	E1359	Y1544	39.8	42.4	37.3	39.8 ± 1.5
5	K1371	E1554	29.8	38.2	30.2	32.7 ± 2.7
6	F1366	V1548	22.6	59.5	14.2	32.1 ± 13.9
7	S1325	Y1544	19.0	28.5	29.2	25.6 ± 3.3
8	D1333	R1583	0.0	71.2	0.0	23.7 ± 23.7
9	K1335	D1587	16.0	13.6	38.6	22.7 ± 8.0
10	K1332	H1588	17.9	30.4	8.9	19.1 ± 6.2
11	Q1367	V1548	0.0	0.0	41.7	13.9 ± 13.9
12	A1327	R1583	0.0	17.0	22.4	13.1 ± 6.7
13	R1334	D1596	0.0	0.0	33.6	11.2 ± 11.2

**Table 2:** The salt bridges on the interface of A1/A2 complex with an average survival ratio exceeding 10%.

No.	A1	A2	Survival rate (%)			Average (mean ± SEM)
			Run 1	Run 2	Run 3	
1	D1323	R1575	69.9	2.6	87.9	53.4 ± 26.0
2	R1334	E1598	64.8	41.6	30.4	45.6 ± 10.2
3	K1371	E1554	39.9	49.8	39.4	43.1 ± 3.4
4	K1335	D1587	28.7	25.5	62.1	38.8 ± 11.7
5	D1333	R1583	0.0	55.0	0.0	18.3 ± 18.3

### 3.3 Impact of mutations on A1/A2-binding affinity and conformational stability

**Figure 3:**
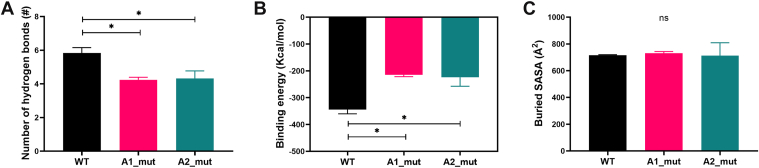
A1_mut and A2_mut complexes exhibited lower binding affinity than the wild-type (WT) complex. (A) Both A1_mut and A2_mut markedly reduced the formation of hydrogen bonds in the A2/A2-binding interface. (B) The mut complexes increased the binding energy of A1/A2. (C) No significant difference was observed in buried solvent accessible surface area (SASA) between the WT and the mut complexes. All data are shown as the mean ± SEM (*n* = 3). One-way anova with Dunnett test for multiple comparisons was used to test statistical significance. ∗*P* < .05; ns, not significant compared with the WT group.

To assess the influence of identified residues on the A1/A2 interaction, we mutated D1323, R1334, and Q1367 on A1 to alanine, creating the A1_mut complex. Similarly, we constructed A2_mut complex with mutations in key residues on A2 (V1546A/R1575A/E1598A). We performed 3 independent 50-nanosecond equilibrium simulations for both mutant complex systems to analyze hydrogen bond formation, binding energy, and buried SASA at the A1/A2-binding interface. The RMSD-Cα and buried SASA time courses for both A1_mut and A2_mut complexes stabilized after 50 nanoseconds, and the distributions of number of hydrogen bonds were fitted to the Gaussian distributions, suggesting these complexes achieved equilibrium (Supplementary Figure 2). In comparison with the WT A1/A2, both A1 and A2 mutations significantly reduced the number of hydrogen bonds at the binding interface (Figure 3A) and increased the binding energy of the A1/A2 complex during equilibration (Figure 3B). The buried SASA were not significantly affected by the mutations in A1 and A2 (Figure 3C). These results imply that the mutations may weaken the binding affinity of A1/A2.

**Figure 4:**
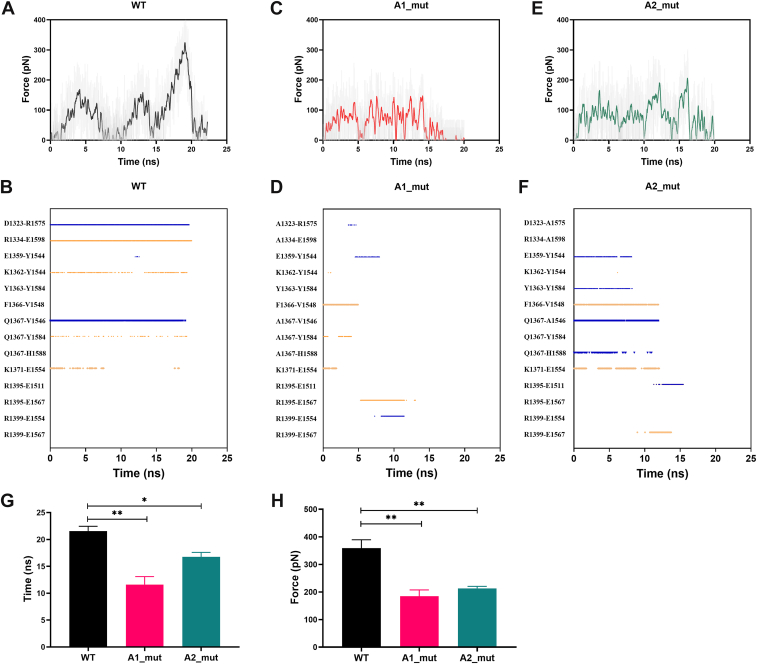
The mechanical stability of the A1_mut and A2_mut complexes was worse than that of the wild-type (WT). Time-dependent loading force profiles for the WT-A1/A2 complex (A), A1_mut complex (C), and A2_mut complex (E). Corresponding interfacial hydrogen bond occupancy patterns at the binding interface of WT (B), A1_mut (D), and A2_mut (F) complexes. Comparison of the average dissociation time (G) and rupture force (H) of the 3 systems. All data are shown as the mean ± SEM (*n* = 3). One-way anova with Dunnett test for multiple comparisons was used to test statistical significance. ns, not significant. ∗*P* < .05; ∗∗*P* < .01 compared with the WT group.

To evaluate the mechanical stability of the A1/A2 interaction, we selected conformations obtained following 50 nanoseconds of equilibrium from the WT, A1_mut, and A2_mut systems as the initial structures for constant velocity stretching simulations. The number of hydrogen bonds, buried SASA, and force curves from the 3 stretching simulations are illustrated in Supplementary Figure 3. Upon dissociation of the A1/A2 complex, both the number of hydrogen bonds and the buried SASA decreased to zero, marking the dissociation time. The peak force observed in the force curve just prior to dissociation was defined as the rupture force. During the stretching simulation, tensions in the WT A1/A2 increased and decreased several times, with a force peak occurring around 20 nanoseconds, followed by a sharp drop leading to dissociation (Figure 4A). Throughout this process, 7 pairs of hydrogen bonds with varying strengths formed at the A1/A2-binding interface. Among these, R1334-E1598, Q1367-V1546, and D1323-R1575 were the strongest pairs, persisting until approximately 20 nanoseconds (Figure 4B), indicating that these residues are essential for maintaining the conformational stability of the A1/A2 complex.

When R1334, D1323, and Q1367 were mutated to alanine, the tension of the A1/A2 complex significantly decreased, resulting in dissociation at approximately 15 nanoseconds (Figure 4C). In the A1_mut system, 8 pairs of hydrogen bonds formed during stretching, but their survival rates were notably low. Specifically, A1334 and A1367 failed to form hydrogen bonds with E1598 and V1546, and the hydrogen bond formed between A1323 and D1575 lasted only 0.52 nanoseconds (Figure 4D). Similarly, mutations of A1598, A1575, and A1546 also induced premature dissociation of the A1/A2 complex (Figure 4E). In comparison with the WT complex, in the A2_mut complex, the hydrogen bonds D1323-A1575, R1334-A1598, K1362-Y1544, and Q1367-Y1584 were absent, and the hydrogen bond Q1367-A1546 was weakened. Instead, hydrogen bond E1359-Y1544 was enhanced, and new hydrogen bonds including Y1363-Y1584, F1366-V1548, Q1367-H1588, R1395-E1511, and R1399-E1567 were formed during this process (Figure 4F). The average dissociation time for WT-A1/A2 was 21.6 ± 0.9 seconds, significantly longer than those of A1_mut (11.6 ± 1.5 seconds) and A2_mut (16.7 ± 0.8 seconds) complexes (Figure 4G). Furthermore, the average rupture force for WT system was 358.7 ± 30.8 pN, notably higher than that of A1_mut (184.4 ± 23.2 pN) and A2-mut (213.2 ± 7.4 pN) complexes (Figure 4H). In conclusion, residue pairs R1334-E1598, Q1367-V1546, and D1323-R1575 are critical for maintaining the mechanical stability of the A1/A2 complex, and both A1_mut and A2_mut weakened its conformational stability and accelerated dissociation.

### 3.4 Key residues regulated A1/A2 interaction

**Figure 5:**
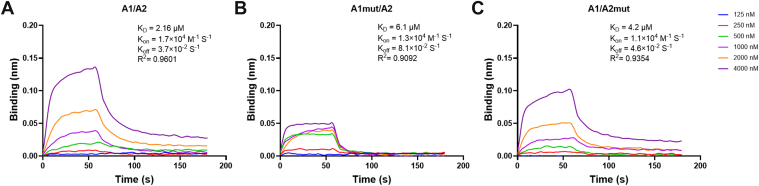
Binding kinetics between recombinant A2 and A1 were measured using biolayer interferometry. (A–C) Representative association and dissociation curves of A2 with various A1 mutants. The experiments were performed in triplicate, with 1 set of data presented as a representative example.

To verify the roles of the hydrogen bonds R1334-E1598, Q1367-V1546, and D1323-R1575 in the A1/A2 interaction, we prepared the VWF-A1 and A2 proteins and their respective mutants and measured the binding kinetics using BLI. In this assay, A2 was immobilized on streptavidin biosensors and exposed to 6 concentrations of purified A1 proteins carrying various mutations. Kinetic analysis showed that WT A1 bound to A2 with a binding affinity (*K*_D_) of 2.16 μM (*K*_on_ = 1.7 × 10^4^ M^−1^s^−1^; *K*_off_ = 3.7 × 10^−2^ s^−1^) (Figure 5A). Mutations of D1323, R1334, and Q1367 on A1 to alanine increased *K*_D_ to 6.1 μM (*K*_on_ = 1.3 × 10^4^ M^−1^s^−1^; *K*_off_ = 8.1 × 10^−2^ s^−1^) (Figure 5B). Similarly, A2_mut weakened the A1/A2 interaction with a binding affinity *K*_D_ of 4.2 μM (*K*_on_ = 1.1 × 10^4^ M^−1^s^−1^; *K*_off_ = 4.6 × 10^−2^ s^−1^) (Figure 5C). These results were consistent with computational simulations.

**Figure 6:**
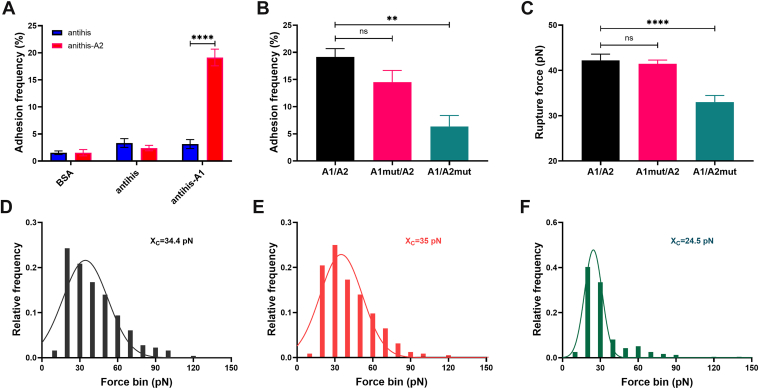
Mutations on A2 significantly attenuate the A1/A2 interaction. (A) The specific binding of von Willebrand factor–A1/A2. (B) The adhesion frequencies of A1/A2, A1mut/A2, and A1/A2mut. (C) The mean rupture force of A1/A2, A1mut/A2, and A1/A2mut. The frequency distribution and Gaussian fitting of the rupture force of A1/A2 (D), A1mut/A2 (E), and A1/A2mut (F). *X*_C_ means the central value of the peak. All data are shown as the mean ± SEM (*n* = 3). One-way anova with Dunnett test for multiple comparisons was used to test statistical significance. BSA, bovine serum albumin. ∗∗*P* < .01; ∗∗∗∗*P* < .0001; ns, not significant compared with the WT group.

To further confirm the impact of these key residues on the interaction between the A1 and A2 domains, we performed AFM experiments. VWF-A1 and VWF-A2 were immobilized on substrates and AFM probes by anti-His mAb, respectively, and the intermolecular adhesion frequency and rupture force were measured by the continuous cyclic contact between the probe and substrate. The adhesion frequencies of the anti-His probe to blank BSA-enclosed substrates, anti-His substrates and the functionalized anti-His–A1 substrates were <5%. However, the anti-His-A2–functionalized probe adhered specifically to the anti-His–A1 substrate with a frequency exceeding 15% (Figure 6A), confirming that the mode of anti-His functionalization did not interfere with specific adhesion measurements between VWF-A1 and VWF-A2. The adhesion frequency between A1_mut and A2 was 14.5% ± 2.1%, slightly lower than that of the WT group (19.1% ± 21.5%), although the difference was not statistically significant. In contrast, the adhesion frequency between A1 and A2_mut was significantly reduced to 6.4% ± 2% (Figure 6B). Similarly, while the average rupture force of WT A1/A2 (42.2 ± 1.4 pN) showed no statistically significant difference from that of the A1_mut/A2 group (41.5 ± 0.8 pN), it was markedly higher than that of the A1/A2_mut group (33 ± 0.5 pN) (Figure 6C). To account for potential outliers, we Gaussian fitted the frequency distribution of all rupture forces to determine the central peak value (*X*_C_). Consistent with the average rupture force trends, the *X*_C_ for the WT group was found to be 34.4 pN (Figure 6D), while that for the A1_mut/A2 group, it was 35 pN (Figure 6E). Notably, the *X*_C_ for the A1/A2_mut group was 24.5 pN (Figure 6F), markedly lower than that of both the WT and A1_mut/A2 groups.

The AFM experiments showed only a nonsignificant weakening trend for A1_mut binding to A2, likely because AFM adhesion frequency assays primarily capture binding events (on-rate) rather than affinity. AFM results were consistent with BLI data, which showed that the *K*_on_ of A1_mut binding to A2 (1.3 × 10^4^ M^−1^s^−1^) was comparable with that of the WT A1/A2 interaction (1.7 × 10^4^ M^−1^s^−1^). Taken together, the BLI and AFM results indicate that residues D1323, R1334, and Q1367 on A1, together with V1546, R1575, and E1598 on A2, are critical for stabilizing the A1/A2 interaction.

## 4 Discussion

VWF is a critical regulatory protein in blood coagulation, and its self-inhibition mechanism is vital for maintaining hemostasis and preventing thrombosis [1]. Under physiological conditions, VWF adopts a self-inhibited, loosely collapsed, globular conformation, wherein the A2 domain shields the GPIbα-binding site on the A1 domain. This configuration effectively prevents the abnormal recruitment and activation of circulating platelets [14]. Recent studies have demonstrated that exogenous A2 fragments ameliorate the coagulation function in posttraumatic brain injury by competitively binding to endogenous VWF-A1 [18], highlighting the therapeutic potential of targeting the A1/A2 interface. However, due to the lack of a complete crystal structure for the VWF-A1/A2 complex, the molecular mechanism governing this interface remains incompletely understood. In this study, we used a combination of HADDOCK molecular docking, MD simulation, and AFM single-molecule force spectroscopy to elucidate the VWF-A1/A2 interaction interface. We demonstrated that this interface comprises a stable hydrogen bond network formed by 3 key interactions: R1334-E1598, Q1367-V1546, and D1323-R1575. Our findings confirmed that residues V1546, R1575, and E1598 play essential roles in maintaining the self-inhibition of A1/A2.

Recent advancements in computational biology have illuminated the interaction mechanism of the VWF-A1/A2 domains. Using molecular docking (PatchDock/FireDock), Aponte-Santamaría et al. [19] identified 6 predominant complex conformations and revealed that the A2 domain sterically occludes the functional β_3_-strand of A1. They proposed 2 primary binding modes: a C-terminal dominant mode (mediated by strong interactions involving R1668) and a β_3_-sheet dominant mode (with D1587 as a core regulatory residue) [19]. Our optimal conformation aligns with the second mode by Aponte-Santamaría et al. [19] but presents distinct features. Although D1587 forms hydrogen bonds and salt bridges with K1335 in our model, its role is not the most critical. Additionally, the HEX-derived model A (the most stable energy state) by Karoulia et al. [20] partially conserves with our model: the 9 key residues involved in the A1/A2 interaction in the model (Q1367, F1366, and K1371 in A1 and V1546, V1548, E1554, R1583, D1587, and H1588 in A2) were all reproduced in our model (Figure 2E). However, E1640 in model B and E1511, E1519, E1522, and D1663 in model C did not appear in our model, suggesting that different docking strategies may capture different binding sites of the VWF-A1/A2 interaction. While these computational frameworks provide valuable mechanistic hypotheses, challenges remain, including intermodel conformational divergence and lack of experimental verification.

Our study identified R1334-E1598, Q1367-V1546, and D1323-R1575 as the 3 most robust hydrogen-bonding pairs at the A1/A2 interface, contrasting with previous research focusing on residues like D1587 or E1554. Notably, mutation Q1367R impairs platelet adhesion under physiological shear stress (300 and 1500 s^−1^), as demonstrated by Cruz et al. using microfluidics [25]. Matsushita et al. [26] found that the R1334A mutation weakens the ristocetin-mediated binding of platelets to VWF. These data collectively confirm that R1334 and Q1367 residue within the GPIbα-binding epitope. Given that A2 binding sterically occludes this functional site [6], we propose that R1334 and Q1367 not only mediate platelet adhesion but also stabilize the A1/A2 complex through the newly discovered hydrogen-bonding network (R1334-E1598 and Q1367-V1546), which may explain how A2 competitively inhibits GPIbα binding through steric occlusion.

To validate the key sites predicted by our computational simulations, we constructed mutant systems (A1_mut: D1323A, R1334A, and Q1367A; and A2_mut: V1546A, R1575A, and E1598A) and conducted multiscale verification by combining MD simulation, BLI, and AFM experiments. In the BLI experiments, A1_mut reduced A1/A2-binding affinity (*K*_D_: 2.16 vs 6.1 μM), primarily due to accelerated dissociation (*K*_off_: 3.7 × 10^−2^ vs 8.1 × 10^−2^ s^−1^), with only a modest effect on association (*K*_on_: 1.7 × 10^4^ vs 1.3 × 10^4^ M^−1^s^−1^). This may explain why, in the AFM experiments—which mainly capture binding events rather than affinity—the A1 mutant showed only a downward trend in adhesion frequency without reaching statistical significance. Notably, in the A2_mut simulations, it was found that this mutation not only significantly reduced the binding affinity of the A1/A2 complex in the equilibrium state but also weakened its mechanical stability. Consistently, BLI and AFM assays with A2_mut confirmed clear disruption of the A1/A2 interaction. These results suggested that residues D1323, R1334, Q1367, V1546, R1575, and E1598 are critical for maintaining the conformational stability of the A1/A2 complex.

The micromolar-range *K*_D_ (2.1 μM) measured by BLI in our study differs from the value reported by Xu et al. (64.9 nM) [18]. The discrepancy may result from variations in experimental methodology, protein sequences, or protein expression systems used in the 2 studies. In contrast to the A2 domain used in our study (residues M1473-G1672, expressed in HEK293 cells), Xu et al. used an *Esherichia coli*–expressed A2 construct (G1481-R1668) that lacks the C-terminal disulfide bond, the absence of which has been reported to enhance A1/A2 interactions. Notably, the micromolar-range *K*_D_ (∼2.1 μM) of A1/A2 is comparable with that of A1-GPIbα interaction [27]. Xu et al. [18] demonstrated that monomeric A2 can bind A1, suppress VWF-mediated platelet hyperadhesion, and ameliorate coagulation abnormalities in a mouse model of traumatic brain injury [18]. This functional evidence strongly supports the biological significance of the A1-A2 interaction.

Database analyses further highlighted their clinical significance. Several of these residues, including D1323, R1334, V1546, R1575, and E1598, are listed in the gomad database. ClinVar databases record their association with von Willebrand disease (VWD). Mutations at V1546 are classified as “likely pathogenic” and may be associated with type 1 and type 2 VWD, while mutations at E1598 are reported as pathogenic or likely pathogenic and linked to type 3 VWD [28]. Currently, no significant pathogenic variants linked to VWD have been reported at D1323, R1334, or R1575 in ClinVar. These findings suggest that V1546 and E1598 may indeed play functional roles in VWD pathogenesis, whereas D1323, R1334, and R1575 appears less critical based on current genetic evidence.

In conclusion, this study provides the first experimental validation that D1323, R1334, and Q1367 on A1 and V1546, R1575, and E1598 on A2 stabilize the A1/A2 complex by forming a strong hydrogen bond network, thereby maintaining A1/A2 self-inhibition. A limitation of this work is that the experiments were performed with isolated A1 and A2 domains. Further studies with full-length VWF will be needed to confirm the physiological relevance of these interactions within the mature multimeric protein. Despite this limitation, these findings advance our understanding of the regulatory mechanism governing VWF function and provide a structural basis for developing therapeutic strategies targeting the A1/A2 interface.
