# Supplementary material for: Key residues regulating von Willebrand factor A1/A2 interactions: insights from molecular dynamics simulations and experimental validation
Source: Res Pract Thromb Haemost. 2025 Nov 17;9(8):103267. doi: 10.1016/j.rpth.2025.103267 (PMC12743552; doi:10.1016/j.rpth.2025.103267)
Supplement: Supplementary Figures 1-3 [file mmc1.docx]

**Supplementary Material**

**Supplementary Figures**


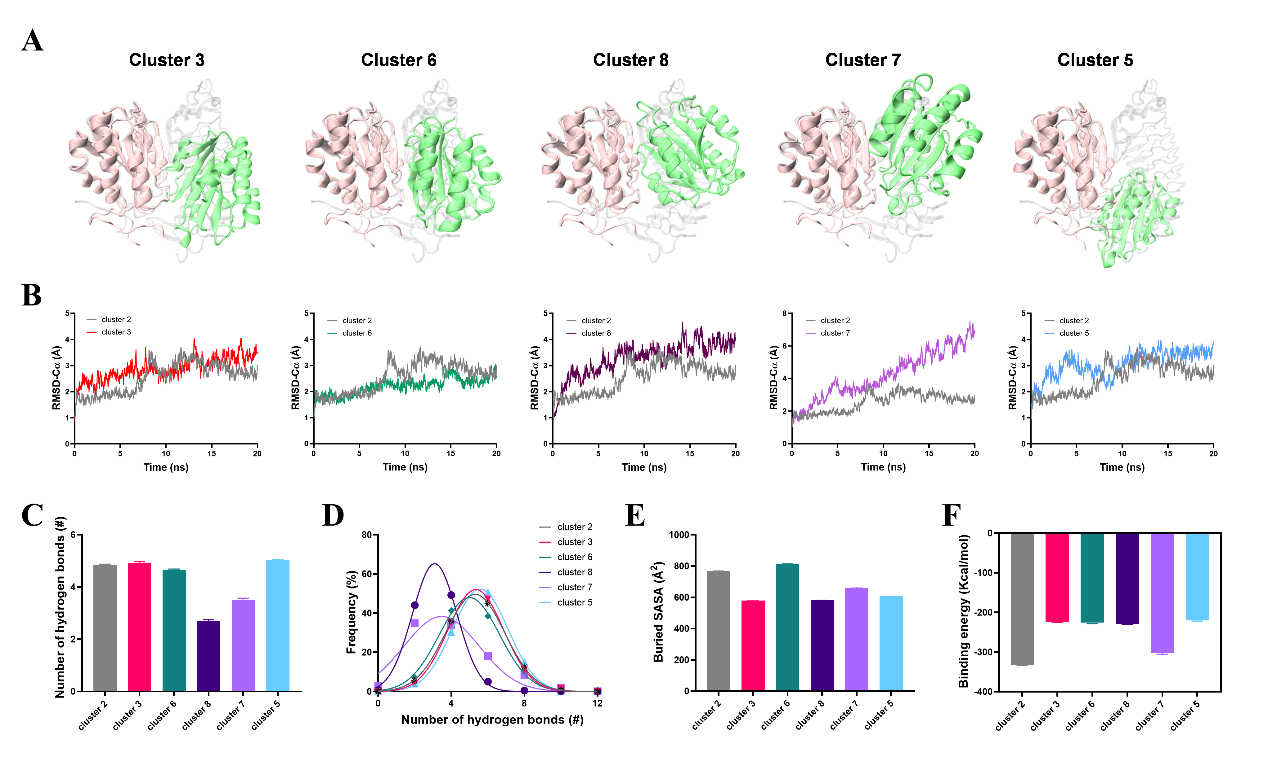


**Fig S1. Stability identification of five docking conformations.** (A) Structure of cluster 3, cluster 6, cluster 8, cluster 7, and cluster 5 obtained from molecular docking. The VWF-A1/A2 complex conformation (orange: VWF-A1 domain; green: VWF-A2 domain) is superimposed on the VWF-A1/GPIbα complex (1SQ0, white base layer). (B) Time courses of RMSD-Cα of these five docking conformations were compared with cluster 2. (C-D) The average number of hydrogen bonds (C) and their distribution (D). (E-F) Buried SASA (E) and binding energy (F) of cluster 3, cluster 6, cluster 8, cluster 7, and cluster 5 during 20 ns equilibrium.


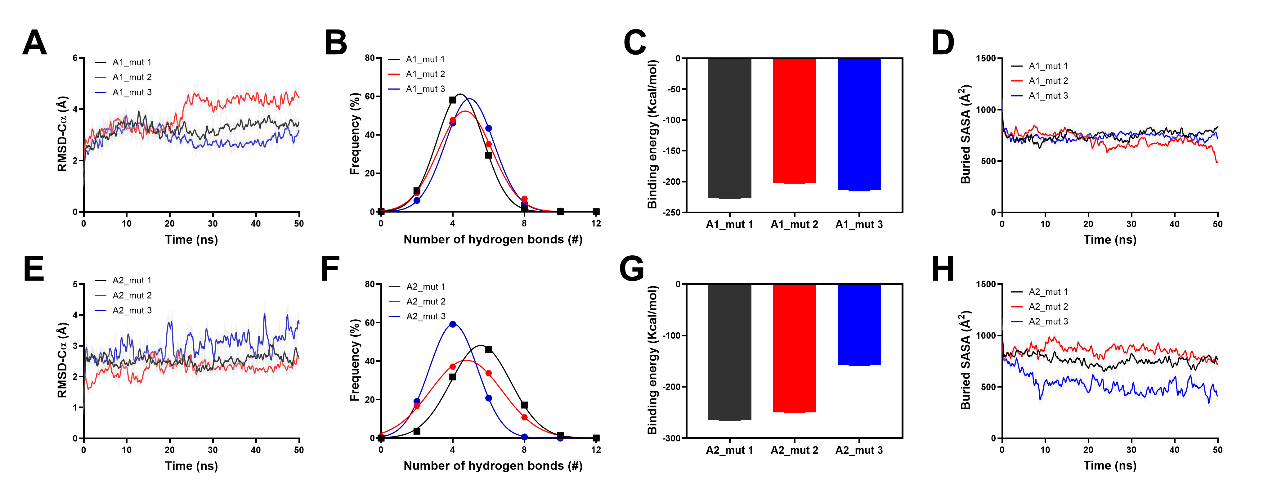


**Fig S2. Variations of structural stability of the A1_mut and A2_mut complexes.** (A and E) Time course of RMSD-Cα of A1_mut (A) and A2_mut (E) complexes from three runs. (B and F) The frequencies of hydrogen bonds number of A1_mut (B) and A2_mut (F) complexes. (C and G) The mean binding energy of A1_mut (C) and A2_mut (G) complexes. (D and H) Time course of buried SASA of A1_mut (B) and A2_mut (F) complexes were showed.


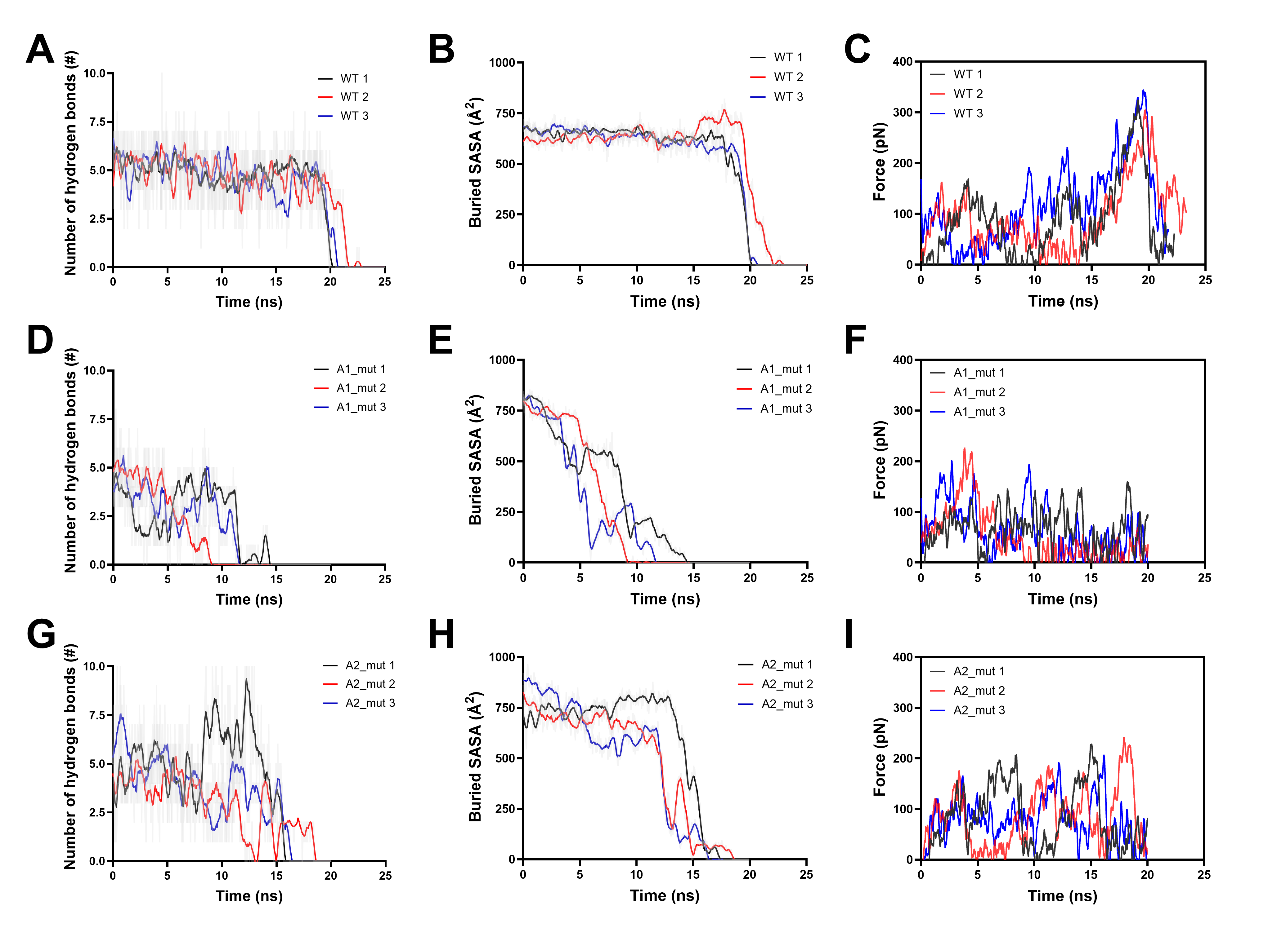


**Fig S3. SMD simulation of WT, A1_mut, and A2_mut complexes.** (A, D, and G) Time course of hydrogen bonds number of WT(A), A1_mut (D), and A2_mut (G) complexes from three SMD simulations. (B, E, and H) Time course of buried SASA of WT(B), A1_mut (E), and A2_mut (H) complexes were showed. (C, F, and I) Time-dependent loading force profiles for the WT(C), A1_mut (F), and A2_mut (I) complexes.
